# Quantitative detection of economically important *Fusarium oxysporum* f. sp. *cubense* strains in Africa in plants, soil and water

**DOI:** 10.1371/journal.pone.0236110

**Published:** 2020-07-20

**Authors:** Megan Ceris Matthews, Diane Mostert, Privat Ndayihanzamaso, Lindy Joy Rose, Altus Viljoen

**Affiliations:** Department of Plant Pathology, Stellenbosch University, Stellenbosch, Western Cape, South Africa; Georg-August-Universitat Gottingen, GERMANY

## Abstract

Banana is an important food crop and source of income in Africa. Sustainable production of banana, however, is at risk because of pests and diseases such as Fusarium wilt, caused by the soil-borne fungus *Fusarium oxysporum* f. sp. *cubense* (Foc). Foc can be disseminated from infested to disease-free fields in plant material, water and soil. Early detection of Foc using DNA technologies is thus required to accurately identify the fungus and prevent its further dissemination with plants, soil and water. In this study, quantitative (q)PCR assays were developed for the detection of Foc Lineage VI strains found in central and eastern Africa (Foc races 1 and 2), Foc TR4 (vegetative compatibility groups (VCG) 01213/16) that is present in Mozambique, and Foc STR4 (VCG 0120/15) that occurs in South Africa. A collection of 127 fungal isolates were selected for specificity testing, including endophytic *Fusarium* isolates from banana pseudostems, non-pathogenic *F*. *oxysporum* strains and Foc isolates representing the 24 VCGs in Foc. Primer sets that proved to be specific to Foc Lineage VI, Foc TR4 and Foc STR4 were used to produce standard curves for absolute quantification, and the qPCR assays were evaluated based on the quality of standard curves, repeatability and reproducibility, and limits of quantification (LOQ) and detection (LOD). The qPCR assays for Foc Lineage VI, TR4 and STR4 were repeatable and reproducible, with LOQ values of 10^−3^–10^−4^ ng/μL and a LOD of 10^−4^–10^−5^ ng/μL. The quantitative detection of Foc strains in Africa could reduce the time and improve the accuracy for identifying the Fusarium wilt pathogen from plants, water and soil on the continent.

## Introduction

Millions of Africans rely on banana (*Musa* spp.) as a staple food and a source of income [1, 2]. The main bananas produced on the continent are cooking bananas such as the East African Highland bananas (EAHB) grown in East and Central Africa (ECA), and the plantains planted in Central and West Africa [1, 3]. Dessert varieties such as Cavendish (AAA), Gros Michel (AAA), Silk (AAB) and Pisang Awak (ABB) bananas are also popular and are consumed as snacks, brewed or exported [4, 5]. Most bananas produced in Africa are grown by small-scale and subsistence farmers in small farms and backyard gardens [1]. Commercial growers produce Cavendish bananas in large plantations, mainly in southern and West Africa, for local markets and export. Factors that limit banana production include diseases such as banana Xanthomonas wilt (BXW), black Sigatoka, banana bunchy top disease (BBTD) and Fusarium wilt [6, 7]. Of these, Fusarium wilt is considered the most devastating.

The causal agent of banana Fusarium wilt, *Fusarium oxysporum* f. sp *cubense* (Foc), is a diverse, anamorphic soil-borne fungus [8]. Foc can be divided into races, vegetative compatibility groups (VCGs), clades and clonal lineages. The races are recognized by their pathogenicity to specific banana cultivars [9]. Foc race 1 causes disease to Gros Michel, Pisang Awak and Silk bananas, Foc race 2 to Bluggoe (ABB) and other cooking bananas, and Foc race 4 affects Cavendish and many varieties susceptible to Foc races 1 and 2. Foc race 4 can be further divided into subtropical race 4 (STR4) and tropical race 4 (TR4) strains [7, 10]. Foc STR4 predominantly causes Fusarium wilt in Cavendish bananas that are subjected to environmental stresses like cold temperatures during winter, whereas Foc TR4 infects Cavendish under both subtropical and tropical conditions [11]. Foc can also be divided into 24 VCGs, which group into eight to nine clonal lineages [12, 13, 14]. Foc race 1 and 2 isolates found in ECA all clustered together in Foc Lineage VI, which includes VCGs 0124, 0125, 0128, 01212, 01220 and 01222 [15]. Foc TR4 and STR4 isolates in Africa include VCGs 01213/16 and 0120, respectively.

It is believed that Foc in ECA was introduced with contaminated plants from Asia, whereas Foc race 1 and 2 isolates in West Africa could have been introduced from contaminated fields in Latin America [5]. Today, all races of Foc are present on the continent. Foc Lineage VI isolates in ECA affect sweet dessert varieties such as Gros Michel and Sukari Ndizi, beer bananas such as Pisang Awak, and cooking varieties such as Mchare (AA) and Bluggoe bananas. The EAHBs and plantains grown in ECA and in West Africa, respectively, are not susceptible to Foc races 1, 2 and STR4. A pilot study on a small selection of EAHB and plantain cultivars also indicated that they are resistant to Foc TR4 [16], but the response of a larger collection of cultivars still needs to be performed. Cavendish bananas, which are also resistant to Foc races 1 and 2, are affected by Foc TR4 (VCG 01213/16) in Mozambique [17] and Foc STR4 (VCG 0120) in South Africa [18]. With the expansion of dessert bananas in Africa for fresh consumption, beer and wine production, and export, there is a significant risk that Foc TR4 may cause significant damage to small and commercial production in future [1, 3, 19].

Foc is a member of the *F*. *oxysporum* species complex [20]. Conventional diagnostics of *F*. *oxysporum* requires lengthy culturing, microscopy and pathogenicity tests. Members of *F*. *oxysporum* can be distinguished from other *Fusarium* species based on their morphological features, but pathogenic and non-pathogenic *F*. *oxysporum* strains are morphologically indistinguishable [21, 22, 13, 23]. Pathogenicity tests can be used to differentiate Foc from other pathogenic members (*formae specialis)* of *F*. *oxysporum* and to distinguish Foc races. Yet, pathogenicity testing is time-consuming and variable environmental conditions may yield inconsistent results [24].

DNA-based diagnostics have improved the speed and accuracy with which plant pathogens are identified. Conventional PCR with species-specific primers is a popular and accurate means of identification, but frequently requires the culturing of pathogens and only provides qualitative results [25, 26]. Quantitative (q)PCR has been developed to address the inadequacies of conventional PCR and can be used for the absolute quantification of pathogen DNA at low concentrations, often directly from environmental samples [27, 28, 29]. Quantitative PCR assays have been developed for Foc race 4 [30] and Foc TR4 [31] in plant samples, but not in soil and water. Markers developed by Lin *et al*. (2013) [30] cannot distinguish Foc TR4 from STR4, and markers developed by Aguayo *et al*. (2017) [31] amplify DNA from the closely related Foc TR4 VCGs 0121 and 01213/16. Loop-mediated isothermal amplification (LAMP) assays have also been developed for the detection of Foc race 4 and TR4 [32, 33].

Foc spores can be dispersed with banana plants, soil attached to shoes, machinery and plantation tools, and with water [8, 34]. Most of the banana growers in Africa are small commercial or subsistence farmers who use suckers rather than tissue culture plants as planting material [1, 15]. If such suckers are infected with Foc, even without showing systems, the fungus can unknowingly be spread to new areas. Foc may remain undetected in newly infested fields for extended periods and its presence may only be noticed when external symptoms appear on susceptible banana plants. Once established in banana fields, Foc spores can also be spread with run-off water that ends up in irrigation systems [8, 35, 36]. The objective of this study was therefore to develop specific and affordable qPCR assays for the detection of Foc Lineage VI, VCG 01213/16 (TR4) and VCG 0120/15 (STR4), which are of economic importance in Africa and globally. This will include primer design, specificity testing, and detection from banana plants, water and soil samples.

## Materials and methods

### Fungal isolates

A total of 127 isolates were used in this study. These included Foc isolates representing all VCGs, lineages and races [12, 14], non-pathogenic *F*. *oxysporum* strains and endophytic *Fusarium* spp. associated with banana pseudostems (Table 1). Non-pathogenic *F*. *oxysporum* isolates were isolated from *Musa* roots and the rhizosphere in Kiepersol, South Africa. All isolates are deposited at the *Fusarium* collection at the Department of Plant Pathology, Stellenbosch University in South Africa.

**Table 1:** *Fusarium* isolates used to develop quantitative PCR assays for *Fusarium oxysporum* f. sp. *cubense* Lineage VI, Tropical Race 4 (TR4) and Subtropical Race 4 (STR4).

CAV number^a^	Alternative culture no.	Species	Foc Lineage/Pathogenicity	VCG	Location
92 ^b c^	-	Foc	Lineage IV	0120	South Africa
180^b c^	Taiwan 14	Foc	Lineage V	0121	Taiwan
181 ^b c^	Phil 36	Foc	Lineage II/IV	0122	Philippines
184 ^b c^	-	Foc	Lineage VI	0125	Australia
185 ^c^	Phil 6	Foc	Lineage I/II	0126	Philippines
186 ^c^	-	Foc	Lineage II/III	0129	Australia
188 ^b c^	STNPZ (RP59)	Foc	Lineage VI	01212	Tanzania
189 ^b c^	RPMW 40	Foc	Lineage VIII	01214	Malawi
191 ^b c^	Indo 160	Foc	Lineage IV	0120/15	Indonesia
193 ^b c^	Mal 6	Foc	Lineage VII	01217	Malaysia
194 ^b c^	Indo 5	Foc	Lineage VII	01218	Indonesia
195 ^b^	Indo 25	Foc	Lineage I/II	01219	Indonesia
202	-	*F*. *oxysporum*	Non-pathogen	-	South Africa
291	C1	Foc	Lineage IV	0120	Canary Islands
300 ^b^	CV-1	Foc	Lineage V	01213	Indonesia
344	CBS 489.97	*F*. *redolens*	-	-	-
346	CBS 680.89	*F*. *oxysporum*	-	-	-
349	MRC 3236	*F*. *anthophilum*	-	-	-
352	MRC 8381	*F*. *avenaceum*	-	-	-
354	MRC 8391	*F*. *chlamydosporum*	-	-	-
367	MRC 1813	*F*. *equiseti*	-	-	-
369	MRC 8532	*F*. *fujikuroi*	-	-	-
372	MRC 4927	*F*. *graminearum*	-	-	-
374	MRC 8544	*F*. *konzum*	-	-	-
386	MRC 8549	*F*. *proliferatum*	-	-	-
388	MRC 8551	*F*. *sacchari*	-	-	-
396	MRC 6715	*F*. *semitectum*	-	-	-
399	MRC 8454	*F*. *solani*	-	-	-
400	MRC 43	*F*. *sporotrichioides*	-	-	-
404	MRC 8554	*F*. *subglutinans*	-	-	-
406	MRC 8558	*F*. *thapsinum*	-	-	-
411	MRC 8560	*F*. *verticillioides*	-	-	-
528	-	*F*. *oxysporum*	Non-pathogen	-	South Africa
529	-	*F*. *oxysporum*	Non-pathogen	-	South Africa
531	-	*F*. *oxysporum*	Non-pathogen	-	South Africa
531	-	*F*. *oxysporum*	Non-pathogen	-	South Africa
532	-	*F*. *oxysporum*	Non-pathogen	-	South Africa
533	-	*F*. *oxysporum*	Non-pathogen	-	South Africa
534	-	*F*. *oxysporum*	Non-pathogen	-	South Africa
535	-	*F*. *oxysporum*	Non-pathogen	-	South Africa
545	-	*F*. *oxysporum*	Non-pathogen	-	South Africa
548	-	*F*. *oxysporum*	Non-pathogen	-	South Africa
551	-	*F*. *oxysporum*	Non-pathogen	-	South Africa
554	-	*F*. *oxysporum*	Non-pathogen	-	South Africa
557	-	*F*. *oxysporum*	Non-pathogen	-	South Africa
561	-	*F*. *oxysporum*	Non-pathogen	-	South Africa
564	-	*F*. *oxysporum*	Non-pathogen	-	South Africa
565	-	*F*. *oxysporum*	Non-pathogen	-	South Africa
566	-	*F*. *oxysporum*	Non-pathogen	-	South Africa
600 ^c^	Indo 16	Foc	Lineage IV	Gen 2*	Indonesia
606 ^b c^	Thai 13	Foc	Lineage VI	0124/5	Thailand
612 ^b c^	RPCR1-1	Foc	Lineage IV	01215	Costa Rica
615 ^c^	PHIL18	Foc	Lineage III	Gen 8*	Phillipines
616 ^c^	PHIL1	Foc	Lineage V	Gen 7*	Phillipines
617 ^c^	23707	Foc	Lineage III	0129/11	Phillipines
620 ^b c^	PHIL24	Foc	Lineage VI	Gen 9*	Phillipines
624 ^c^	PHIL26	Foc	Lineage VI	Gen 10*	Phillipines
629 ^b^	22468	Foc	Lineage VI	0125	Australia
632 ^b c^	RP53	Foc	Lineage I/II	01210	USA
785 ^b c^	RP JAK 4	Foc	Lineage I/II	0126	USA
789 ^b c^	25111	Foc	Lineage V	01213/16	Australia
818 ^b c^	Indo 54	Foc	Lineage IV	Gen 3*	Indonesia
849	Indo 57	Foc	Lineage I/II	01219	Indonesia
853 ^b^	Mal 4	Foc	Lineage VI	Gen 4*	Malaysia
893 ^b c^	23997	Foc	Lineage VI	0128	Australia
898 ^b c^	Phil 4	Foc	Lineage VI	Gen 11*	Philippines
909 ^b c^	Mal 65	Foc	-	Gen 5*	Malaysia
997 ^c^	Tanz 9	Foc	Lineage VI	0124/5/8	Tanzania
1003 ^b c^	Viet 1	Foc	-	Gen 13*	Vietnam
1004 ^b c^	Viet 3	Foc	Lineage VI	Gene 14*	Vietnam
1011 ^b c^	Viet 10	Foc	-	Gen 12*	Vietnam
1024 ^b^	Mex 2	Foc	Lineage VI	Gen 6*	Mexico
1036 ^b c^	RP 2	Foc	Lineage VII	0123	Philippines
1089 ^b^	N5447	Foc	Lineage III	0129	Australia
1563	-	Foc	Lineage V	01213/16	Oman
1683	-	Foc	Lineage V	01213/16	Philippines
1703	-	*Fusarium* spp.	-	-	Philippines
1824	-	*F*. *oxysporum*	Non-pathogen	-	Uganda
1830	-	*Fusarium* spp.	-	-	Philippines
1833	-	Foc	-	-	Philippines
1842	-	Foc	Lineage I/II	0126	Philippines
1897	-	Foc	Lineage VI	0125	India
1905	-	*Fusarium* spp.	-	-	India
1907	-	*Fusarium* spp.	-	-	India
1910	-	Foc	-	-	India
1912	-	Foc	Lineage VI	0125	India
1927	-	Foc	Lineage VI	-	India
1933	-	Foc	Lineage VI	-	India
1960	-	Foc	Lineage VI	-	Sri Lanka
1970	-	Foc	Lineage VI	-	Sri Lanka
1974	-	Foc	Lineage VI	-	Sri Lanka
2030	-	*Fusarium* spp.	-	-	Bangladesh
2031	-	*Fusarium* spp.	-	-	Bangladesh
2107	-	*Fusarium* spp.	-	-	Cambodia
2151	-	*Fusarium* spp.	-	-	Cambodia
2154	-	*Fusarium* spp.	-	-	Cambodia
2251	-	Foc	Lineage VI	0124	Vietnam
2266	-	Foc	-	-	Sri Lanka
2267	-	Foc	-	-	Sri Lanka
2270	—	*Fusarium* spp.	-	-	Malaysia
2271	-	Foc	-	-	Malaysia
2281	-	Foc	-	-	Malaysia
2287	-	Foc	-	-	Malaysia
2351	-	Foc	-	-	Taiwan
2354	-	*Fusarium* spp.	-	-	Taiwan
2400 ^b c^	-	Foc	Lineage VI	01220	Bangladesh
2407	-	Foc	Lineage VI	-	Bangladesh
2413	-	Foc	Lineage VI	-	Bangladesh
2443	-	*Fusarium* spp.	-	-	Malaysia
2611	-	*F*. *oxysporum*	Non-pathogen	-	Tanzania
2633	-	*F*. *oxysporum*	Non-pathogen	-	Tanzania
3049 ^b c^	-	Foc	Lineage V	01213/16	Mozambique
3127	-	Foc	-	-	Phillipines
3128	-	Foc	Lineage VI	-	Phillipines
3130	-	Foc	Lineage VI	-	Phillipines
3142	-	Foc	Lineage VI	-	Philippines
3143	-	Foc	Lineage VI	-	Philippines
3326 ^b c^	-	Foc	Lineage V	01213/16	Jacaranda
3351	-	Foc	-	-	Philippines
3371	-	Foc	-	-	Philippines
3372	-	Foc	Lineage V	01213/16	Philippines
3475	-	Foc	Lineage VI	-	Philippines
3478	-	Foc	Lineage VI	-	Philippines
3481	-	Foc	Lineage VI	-	Mauritius
3484	-	Foc	Lineage VI	-	India
3534	-	Foc	-	-	Indonesia
3540	-	Foc	-	-	Indonesia
-	NRRL 36115 ^b c^	Foc	-	01224	Malaysia
-	NRRL 36116 ^b c^	Foc	-	01223	Malaysia
-	NRRL 36117 ^b c^	Foc	Lineage VI	01222	Malaysia
-	NRRL 36118 ^b c^	Foc	Lineage VII	01221	Thailand

Genotypes that are not compatible to known *Fusarium oxysporum* f. sp. *cubense* (Foc) VCGs as described by Bentley et al. (1998) are indicated with “*”, unavailable data is indicated with ‘‘-”.

^a^Culture collection housed at the Department of Plant Pathology, Stellenbosch University, South Africa.

^b^Isolates that were used to infect plant samples.

^c^Isolates that were used to infect water and soil samples.

^bc^Isolates that were used to infect plant, water and soil samples.

### Preparation of Foc-contaminated samples

Tissue cultured-derived, 10-cm Cavendish and Gros Michel plantlets were each inoculated with 44 Foc isolates representing all VCGs (Table 1), as described by Viljoen *et al*. (2017) [37]. Foc VCG 0120 isolate CAV 179 was used as a positive control, and water as a negative control. Plants were incubated in the greenhouse facilities at the National Quarantine Station of the Department of Agriculture and Fisheries in Stellenbosch, South Africa at 25°C with a 12-hr light cycle. Disease development was determined 6 weeks after inoculation by assessing the yellowing of leaves and discolouration of the inner rhizome. Plants inoculated with water only were used as a source of background DNA for qPCR matrix-matched standard curves.

Water collected from the Coetzenberg dam in Stellenbosch, South Africa, was inoculated with spores representing all Foc VCGs. The spores were first produced in Armstrong media [21] in CELLSTAR® culture flasks (Greiner bio-one Gmbh, Frickenhausen, Germany) that were rotated at 100 x g in a Labcon incubation shaker (Labcon®, Petaluma, California, USA) at room temperature for 2 weeks. Each spore suspension was thereafter filtered through sterile cheesecloth and washed with autoclaved dH_2_O (20 mL) before being centrifuged at 4 000 x g for 2 min in an Eppendorf® 5810 R Centrifuge (Eppendorf AG, Hamburg, Germany). The supernatant was discarded, and the pellet dissolved in the dam water. Dam water was selected to simulate an environmental sample. Spore concentrations were determined and adjusted to ~10^5^ spores mL^-1^. Uninoculated water samples were used as negative controls and as a source of background DNA for qPCR matrix-matched standard curves.

Foc-infested soil samples were prepared by autoclaving 3 kg soil from a banana plantation in South Africa. After autoclaving, 1 g of soil was plated in triplicate onto PDA containing 40 mg L^-1^ streptomycin sulphate to ensure that no viable *F*. *oxysporum* spores were present in the soil. The autoclaved soil was transferred into Magenta^TM^ boxes (Magenta LLC, Lockport, Ilinois, USA), and each box (100 g soil) inoculated with an Foc isolate representing a different VCGs, which was grown on 5 g of millet seed [38]. The inoculated soil was incubated for 6 weeks at room temperature before use. Uninoculated soil samples were used as negative controls and as a source of background DNA for qPCR matrix-matched standard curves.

### DNA extractions

Total genomic DNA (gDNA) from 127 pure fungal cultures (Table 1) were extracted for initial specificity testing after growing the cultures on potato dextrose agar (PDA) in 90-mm-diameter Petri dishes at 25°C for 7 days. Mycelia were scraped off the media with sterile scalpel blades, and transferred into 2-ml Eppendorf tubes. DNA was extracted using the Wizard SV Genomic DNA Purification Systems kit according to the manufacturer’s instructions (Promega, Madison, USA). The quality and quantity of all the DNA samples was evaluated with a NanoDrop™ spectrophotometer (Thermo Fischer, Waltham, Massachusetts, United States).

DNA from the infected banana rhizomes was obtained using an optimised protocol for Nucleospin® Plant II miniprep extraction kits. The rhizomes were first lyophilised with liquid nitrogen, and ~100 mg of the lyophilised material was mixed with 350 μL lysis buffer (Nucleospin® PL2) and 30 glass beads in a 1.5-mL Eppendorf^TM^ tube (Eppendorf, Hamburg, Germany). The tube was then placed in a tissue lyser for 5 min at a frequency of 30/s. After lysis, the DNA extraction was performed as per the manufacturer’s guidelines up to the elution step. During elution, 30 μL buffer (Nucleospin® PE) was added instead of 50 μL.

DNA from 1-mL Foc-infected water samples were extracted using AMPure XP beads (Beckman Coulter, Indianapolis, Indiana, USA) and a DynaMagTM-96 side magnetic plate (Thermo Fischer, Waltham, Massachusetts, USA). The samples were centrifuged at 13 200 x g for 2 min in 1.5-mL Eppendorf tubes, and the supernatants discarded. Glass beads (~10) and CTAB lysis buffer (Nucleospin® PL1) were then added to each tube, and the tubes placed in a tissue lyser for 1 min at a frequency of 30/s, where after they were incubated in a water bath at 65°C for 30 min. The samples were then centrifuged at 13 200 x g for 2 min, and 35 μL supernatant transferred to a single 200-μL tube (Nolato Treff AG, Degersheim, Switzerland). DNA was then extracted from the supernatant according to the AMPure XP manufacture guidelines.

DNA from soil samples was extracted using an optimised protocol for Nucleospin® Soil extraction miniprep kits. The soil samples were first lyophilised with liquid nitrogen, and 1 g per sample was then transferred to 15-mL Falcon tubes (NEST®, New District, Wuxi, Jiangsu, China). Glass beads and 2 mL extraction buffer (10 mL of 0.5 EDTA pH 8, 10 mL of 1 M Tris pH 8, 16.6 mL of 3 M NaCl, 0.7 mL of beta mercaptoethanol, 1.25 mL of 20% SDS made up to 100 mL with autoclaved dH_2_O) were added to each Falcon tube. The tubes were then vortexed for 1 min and incubated at 65°C for 1 hr, with 1 min of vortexing every 15 min. Each 15-mL tube was centrifuged at 4 000 x g for 1 min in an Eppendorf centrifuge (Hamburg, Germany), and 450 μL clear supernatant transferred to 2-mL Eppendorf™ tubes with ceramic beads. DNA was then extracted according to the manufacturer’s guidelines until the elution step, which used 30 μL of elution buffer instead of 50 μL.

### Primer development

#### Selection of target-specific regions

Three different approaches were used to design primers in this study (S1 Fig). In the first approach, a collection of whole genome sequences of 87 isolates representing the 24 known Foc VCGs available at Stellenbosch University were used. DNA of isolates were first sequenced on the Illumina platform, and the data pre-processed with Trimmomatic [39] to filter low quality sequences. Trimmed reads were then mapped to 50 conserved single-copy orthologous genes of the Foc II5 (isolate 54006) reference genome, selected in consideration of [40] and personal communication with Li-Jun Ma (University of Massachusetts, MA, USA). Mapping was done in CLC genomics workbench version 11 (Qiagen, Hilden, Germany) using default parameters to create a consensus sequence of each gene for each isolate. Multiple sequence alignments of all 50 gene areas were performed using MAFFT software version 5.85 [41] and visualized with MEGA software version 7.0.18. [42], to identify target-specific single nucleotide polymorphisms (SNPs), insertions or deletions. SNPs specific to Foc Lineage VI and STR4 (VCG 0120/15) isolates were identified in the DNA-directed RNA polymerase III subunit beta (RPC2) (S1 Appendix) and the Foc II5 FOIG 03031 hypothetical protein (S2 Appendix), respectively.

In the second approach, DNA of 85 different Foc isolates representative of the diversity in Foc were send for diversity array technology sequencing (DArT-seq) (http://www.diversityarrays.com/). The data were analyzed using DArTsoft v.7.4.7 (DArT P/L, Canberra, Australia). The presence or absence of DArTseq alleles and SNPs within these alleles, were then scored as binary data (1/0) and its genomic position indicated. This binary database in Microsoft Excel was then queried to find Foc TR4-specific DArT-seq alleles or SNPs within alleles. The Foc TR4-specific alleles were expanded to include up- and downstream sequences when mapped to the Foc II5 reference genome. This larger region were screened against the whole genome database available at Stellenbosch University and on basic local alignment search tool (BLAST) on NCBI (https://blast.ncbi.nlm.nih.gov/). Only Foc TR4-specific regions were considered for further characterization. The selected target region was a non-coding region within the Foc II5 Supercontig KK036133 on the fungal Ensembl database (https://fungi.ensembl.org/Fusarium_oxysporum_f_sp_cubense_tropical_race_4_54006_gca_000260195/Info/Index). To confirm the sequence identity of the selected target region, it was send for sequencing at the Central Analytical Facility (CAF) of Stellenbosch University (S3 Appendix).

The third approach attempted to find a region common among all Foc isolates. The *secreted-in-xylem* (*SIX)9* gene region was selected as it was reported to be present in all Foc strains [43]. Isolates representative of 24 known Foc VCGs were then sequenced at CAF, Stellenbosch University and a multiple-sequence alignment was created in MAFFT software version 5.85 and visualized with MEGA software version 7.0.18. *SIX9* sequences found on Genbank were also included in the sequence alignment. The database was then manually inspected to identify Foc-specific regions (S4 Appendix).

#### Primer design

Primers were manually designed so that target-specific regions were on the 3’ side of oligonucleotides. The primers were screened with Primer3 software [44] to ensure superior qPCR oligonucleotide properties, including melting temperatures between 55 and 65°C, primer lengths between 18 and 25 bp, GC content between 40 and 60%, no occurrence of primer- or self-dimers, and amplicon sizes of between 90–200 bp.

### PCR conditions and primer specificity

DNA extracted from pure culture Foc isolates that represent the eight Foc lineages described by Fourie *et al*. [14] were used for the initial screening of primer suitability in qPCR. Conditions were optimised for the respective primer sets using annealing temperatures of 60–70°C, two different chemistries (SYBR no-Rox Sensimix or SensiFAST, Bioline, Fremont, California, USA) and a final primer concentration of 0.2–0.4 μM. The qPCR analysis was performed using a Rotor-Gene™-6000 machine (Bio-Rad, Hercules, California, USA). Conditions that resulted in the highest fluorescence of target DNA were selected for subsequent qPCRs to determine the specificity of each assay for the detection of Foc Lineage VI, TR4 and STR4.

To validate the specificity of qPCR markers, a larger set of DNA extracted from 127 pure culture *Fusarium* isolates (Table 1) were used. For each qPCR sample, 10–20 ng/μL DNA, 10 μL SYBR no-Rox Sensimix or SensiFAST (both contain heat-activated DNA polymerase, ultra-pure dNTPs, SYBR Green I dye, stabilizers and enhancers including 6mM MgCl_2_) and 0.6 μL of the forward and reverse primers at optimised concentrations were used in a total reaction volume of 20 μL. The conditions used with SYBR no-Rox Sensimix included an initial denaturing step of 10 min, followed by 40 amplification cycles of 10 s at 95°C, 15 s at the annealing temperature, and 20 s at 72°C. The conditions used with SYBR no-rox SensiFAST included an initial denaturing step of 3 min followed by 40 amplification cycles of 5 s at 95°C, 10 s at the annealing temperature, and 20 s at 72°C. Corbett-type Strip tubes (0.1 mL) and caps (SSIBio, Lodi, California, USA) were used in all qPCR reactions. Specificity of the qPCR was assessed via melt curve analysis (temperature gradients from 72–95°C, with a hold for 90 s on the first step and 5 s on the next steps).

### Optimisation and sensitivity

#### Tests for linearity and the presence of inhibitors

Standard curves were prepared using four-fold dilutions of Foc DNA for Lineage VI, TR4 and STR4 in a fixed background of DNA from Foc-free plant, water and soil samples. Dilution points were used in triplicate qPCR reactions in a Rotor-Gene™-6000 (Bio-Rad, Hercules, California, USA), with the optimised qPCR conditions. Rotor-Gene™ Q-series software was used to plot the cycle threshold (Ct) values of the dilution points against a logarithm of the initial DNA concentration. Standard curves were acceptable if the efficiency was close to 1.00 and the correlation coefficient (R2) was above 0.99 [28].

The concentration of Foc target DNA extracted from plant, water and soil samples, as estimated with qPCR, was compared before and after inhibitor removal. NucleoSpin® Gel and PCR Clean-up kits (Machel-Nagel, Düren, Germany) were used for inhibitor removal. Inhibition is less prominent with dilutions and can be indicated by low amplification efficiencies along a DNA dilution series (like a standard curve) [28]. For plant, soil and water assays, standard curve efficiencies were assessed to ensure inhibitors in the environmental samples did not affect DNA amplification.

#### Reproducibility and repeatability

DNA extracted from Foc-infected plant, water and soil samples were used to test the reproducibility and repeatability of the DNA extraction methods and qPCR assays. DNA was extracted on two separate days from four samples, each infected with a different Lineage VI isolate, in triplicate, to assess inter-sample repeatability and reproducibility, and from one sample six times to determine intra-sample repeatability and reproducibility. The extracted DNA was used in an optimised Lineage VI qPCR assay, and standard deviations recorded between average DNA concentrations within sub-samples and across days. SAS® version 9.4 (SAS Institute Inc., Cary, North Carolina, USA) was used for Leven’s test for homogeneity, Shapiro Wilk’s test for normality, and ANOVA to check if significant differences in average DNA concentration occurred between days as there is no threshold for DNA extraction standard deviations.

To test the reproducibility (inter-assay variation) and repeatability (intra-assay variation) of each qPCR assay, DNA from one sample was used in three or six technical repeats in two different qPCR runs. Standard deviations between average Ct values across qPCR runs and between technical repeats with the highest and lowest mean Ct values were recorded. Standard deviations between replicate Ct values below 0.35 were considered acceptable [45].

#### Limit of quantification and limit of detection

The dilution point below which positive samples cannot be reliably quantified is called the limit of quantification (LOQ), and the dilution point below which positive samples cannot be detected with certainty is called the limit of detection (LOD) [28, 46]. A ten-time dilution series was prepared for Lineage VI, TR4 and STR4 by diluting target DNA in DNA from Foc-free plant, water or soil. The undiluted DNA concentration of Foc was 20 ng/μL and 5 ng/μL for plant and soil, or water backgrounds, respectively. Background DNA consisted of ~20 ng/μL of DNA extracted from a Gros Michel rhizome, ~1.5 ng/uL of DNA extracted from banana plantation soil and ~1 ng/uL of DNA extracted from dam water. DNA from each dilution point was used in triplicate in an optimised qPCR assay. Standard deviations between the Ct values of technical replicates were recorded. A discreet threshold approach was applied to determine the LOQ and LOD for the developed assays. The LOQ was the lowest standard concentration of template DNA that produced positive replicates at a 95% confidence level and a standard deviation below 0.35 between Ct values. The LOD was the lowest standard concentration of template DNA that produced a positive result, however, the standard deviation between technical replications was above 0.35.

### Quantitative PCR of inoculated plant, water and soil samples

The optimised Foc Lineage VI, TR4 and STR4 multiplex qPCRs were used to quantify DNA extracted from artificially inoculated plant, water and soil samples (Table 1). For each qPCR assay, a non-template control and positive DNA of a known concentration were analysed in triplicate qPCR tubes, while extracted DNA was analysed in duplicate.

### Quantitative PCR of naturally infected plant material

Banana pseudostem samples naturally infected with Foc Lineage VI or TR4, of which VCG identity was previously confirmed, were used for testing. DNA was extracted from the symptomatic plant material as described previously and tested in duplicate with Foc Lineage VI and TR4 primers to determine if the qPCR assays could quantify the target pathogen DNA within naturally infected plant samples.

## Results

### Primer design

The number of primer sets designed to target Foc Lineage VI, TR4 and STR4 was 3, 11 and 15, respectively. Seven primer sets were also designed to target all Foc isolates. The top performing primer set selected for Lineage VI was RTLinVI_F3 and FocLinVI-R, for TR4 was RT_13.16_F2.5 and RT_13.16_R2.5, and for STR4 was 0120_15_F7 and 0120_15_R4 (Table 2). Foc_F2 and Foc_R1 primers specific to Foc were selected for combination in a multiplex qPCR with the STR4 primer set. Foc Lineage VI and Foc STR4 specific primers amplified a 98-bp region within the DNA-directed RNA polymerase III subunit beta (RPC2) and a hypothetical protein (Foc II5 FOIG 31310), respectively. Foc TR4-specific primers amplified a 157-bp non-coding region at position kk036133: 22108–22265 of the Foc reference isolate’s (II5) genome. A 198-bp region of the *SIX9a* homolog was amplified by Foc-specific primers (S2 Fig).

**Table 2:** Primer names, sequences and target *Fusarium oxysporum* f. sp. *cubense* (Foc) groups.

Forward primer		Reverse primer		
Name	Sequence	Name	Sequence	Target
Foc_F2	5’-AGTCGGTTGCTACGCTGTC-3’	Foc_R	5’-GAAGCCCAGTTGTAAGGATGA-3’	All Foc isolates
RTLinVI_F3	5’-GACATTTGACGACTTTCTGA-3’	FocLinVI-R	5’-GTGTCACTTGGTCCTCGTAT-3’	Foc Lineage VI
RT_13.16_F2.5	5’-GAATATAAAGAGGAAGTAGCCG-3’	RT_13.16_R2.5	5’-CCTCGCTGAATTATATCTAAACC-3’	Foc TR4
0120_15_F7	5’-CAGTCGAGAACACTCAAGGTTTC-3’	0120_15_R4	5’-ACCGTGTTATCGAGGAGGGA-3’	Foc STR4

### Primer specificity

**Fig 1:**
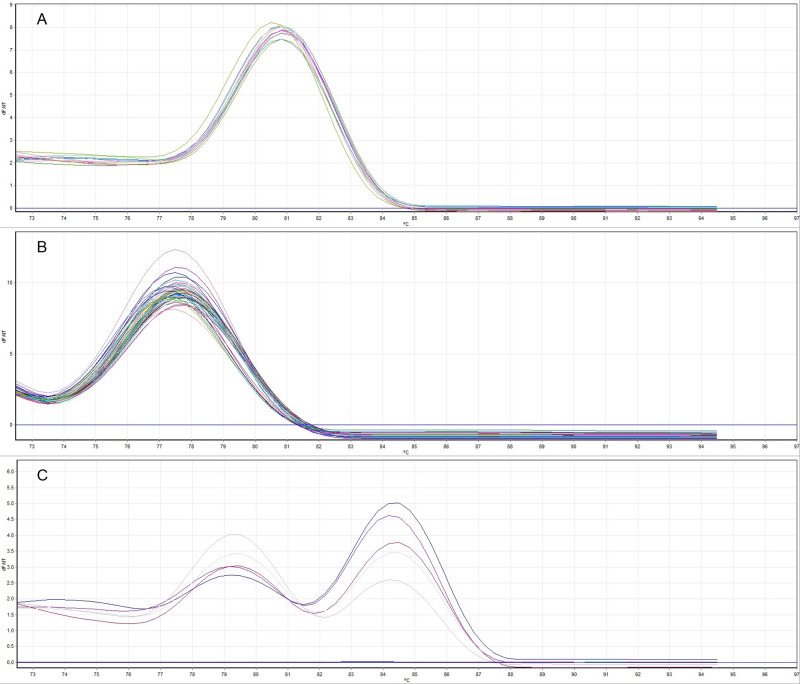
A. Melt curve analysis of 12 *Fusarium oxysporum* f. sp. *cubense* Lineage VI target DNA samples with melting points of 80.2–80.5°C. B: Melt curve analysis of 50 TR4 target DNA samples with melting points of 77.2–77.8°C. C: Melt curve analysis of five STR4 DNA samples with melting points of 79.2–79.5° and 84.2–84.5°C. Target DNA samples varied from ~20–0.02 ng/μl.

The Foc Lineage VI primers RTLinVI_F3 and FocLinVI-R performed best with SYBR Sensimix chemistry at a 66°C annealing temperature and were specific for the detection of Foc Lineage VI isolates. The Foc TR4 primers RT_13.16_F2.5 and RT_13.16_R2.5 performed best with SYBR SensiFAST chemistry, at a 62°C annealing temperature, and were specific in detecting Foc TR4 isolates. Foc STR4 primers 0120_15_F7 and 0120_15_R4 performed best at a 68°C annealing temperature with SYBR Sensimix chemistry but also amplified some non-target isolates, including a *F*. *proliferatum* (CAV 386), *F*. *sacchari*, (CAV 388) and two non-pathogenic *F*. *oxysporum* isolates. When the Foc STR4 primers were combined with Foc-specific primers in a multiplex qPCR assay at a 68°C annealing temperature, the DNA of non-target isolates was not amplified by the Foc specific primers and no peak was present for this amplicon in post-amplification melt analysis. The non-target samples could therefore be eliminated. The STR4 primers were therefore used in multiplex with the Foc primers for further testing and optimisation. The Lineage VI and TR4 primer sets worked best with 0.3-μM primer concentrations. For the STR4 multiplex, concentrations of 0.2 and 0.4 μM were used for Foc_F2 and Foc_R1 and 0120_15_F7 and 0120_15_R4, respectively. The melting points of target amplicons in the optimised qPCR assays were 80–80.5°C (Fig 1A), 77.2–77.8°C (Fig 1B) and 79.2–79.5° and 84.2–84.5°C (Fig 1C) for Lineage VI, TR4 and the STR4 multiplex, respectively.

### Optimisation and sensitivity

#### Tests for linearity and the presence of inhibitors

Inhibitor removal kits did not improve DNA quality, and DNA amplification efficiencies were close to 100%. This indicated that there was no significant inhibition of DNA amplification by the plant, soil and water backgrounds. Standard curves of all the qPCR assays had acceptable efficiencies and R^2^-values close to 1.00, indicating that they were suitable for quantitative detection (Table 3).

**Table 3:** Standard curve parameters for *Fusarium oxysporum* f. sp. *cubense* Lineage VI, TR4 and STR4 quantitative detection in plant, water and soil samples.

Standard Curve		R^2a^	Efficiency^b^	[DNA] range^c^	Ct range^d^	Slope^e^
Plant^f^	Lineage VI	0.99584	0.98	4.62–0.0002 ng/μL	19.22–33.11	3.380
	TR4	0.99672	1.00	4.80–0.0002 ng/μL	18.59–32.42	3.333
	STR4	0.99027	1.00	33.25–0.0012 ng/μL	9.45–24.32	3.318
Water	Lineage VI	0.99546	0.99	4.46–0.0170 ng/μL	17.64–25.72	3.343
	TR4	0.99204	1.03	2.60–0.0100 ng/μL	20.17–27.95	3.252
	STR4	0.99015	1.03	1.45–0.0061 ng/μL	19.84–27.51	3.252
Soil	Lineage VI	0.99030	1.00	5.81–0.0003 ng/μL	19.71–32.95	3.312
	TR4	0.99660	0.98	1.26–0.0003 ng/μL	18.23–30.18	3.382
	STR4	0.99151	1.00	6.43–0.00021 ng/μL	18.49–33.45	3.332

^a^The correlation coefficient (R^2^) of the standard curve representing linearity.

^b^The efficiency (E = (10^(-1/slope)^) -1) of the standard curve.

^c^The range of DNA concentrations within the standard dilution series.

^d^The range of cycling thresholds (Ct) within the standard dilution series.

^e^The slope of the standard curve (M-value).

^f^The environmental background of the standard curve.

#### Reproducibility and repeatability

There is no threshold for DNA extraction repeatability and reproducibility, but standard deviations were included in this study (S1 and S2 Tables). Levene’s test for homogeneity and Shapiro Wilk’s test showed the data to be homogenous and normally distributed (data not presented). No significant differences (P<0.5) were found between the DNA extraction methods used on two different days, indicating that DNA extractions from plant, soil and water was reproducible. Significant differences were found among Foc Lineage VI isolates when DNA was extracted from plant and soil samples, but not when DNA was extracted from the water as Foc inoculum in water was standardised (10^5^ spores/mL) prior to DNA extraction (S3 Table). Standard deviations between target DNA quantified in separate qPCR assays (reproducibility), and within the same qPCR assay (repeatability), did not exceed 0.35 (S4 and S5 Tables). This indicated that all the Foc Lineage VI, TR4 and STR4 qPCR assays were reproducible and repeatable.

#### Limit of quantification and limit of detection

The LOQ and LOD in plant and soil was ~10^−4^ and ~10^−5^ ng/μL for Foc Lineage VI, TR4 and STR4 qPCR assays (Table 4). In water samples the LOQ and LOD was ~10^−3^ and ~10^−4^ ng/μL for all qPCR assays.

**Table 4:** Limit of detection and limit of quantification of *Fusarium oxysporum* f. sp. *cubense* in plant, water and soil samples.

		Ave [DNA] (ng/μL) ^a^		Ave Ct^c^		SD_Ct_^d^	
		LOQ	LOD	LOQ	LOD	LOQ	LOD
Plant^b^	Lineage VI	2.29 x 10^−4^	8.00 x 10^−5^	33.84	35.42	0.159	0.466
	TR4	5.99 x 10^−4^	5.98 x 10^−5^	31.82	35.24	0.194	0.683
	STR4	3.05 x 10^−4^	1.32 x 10^−5^	31.31	33.81	0.40	0.51
Water	Lineage VI	3.48 x 10^−3^	1.40 x 10^−4^	32.35	36.82	0.37	1.00
	TR4	1.05 x 10^−3^	4.50 x 10^−4^	28.79	32.79	0.09	1.13
	STR4	5.05 x 10^−3^	7.50 x 10^−4^	34.16	36.28	0.10	1.53
Soil	Lineage VI	4.86 x 10^−4^	9.43 x 10^−5^	33.32	36.02	0.327	0.762
	TR4	1.64 x 10^−4^	2.98 x 10^−5^	30.98	33.00	0.153	1.879
	STR4	2.20 x 10^−4^	4.90 x 10^−5^	33.50	35.52	0.09	0.46

Ct values–Cycle threshold values according to qPCR analyses.

LOD—Limit of detection.LOQ—Limit of quantification.

^a^The average DNA concentration at the LOQ or LOD.

^b^The environmental sample type (plant/water/soil) that was infected with the target Foc isolates (Lineage VI/TR4/STR4).

^c^Average Ct (cycle threshold) values obtained from three technical replicates of the LOQ/LOD.

^d^The standard deviation between Ct values at the LOD and LOQ.

### Quantitative PCR of inoculated plant, water and soil samples and naturally infected plant material

Foc Lineage VI, TR4 and STR4 qPCR assays were specific when tested with DNA from Foc-infected plant, water and soil samples (Table 1). The Lineage VI and TR4 qPCR assays were also able to detect and quantify target pathogen DNA from naturally infected banana plants (Table 5).

**Table 5:** Quantitative detection of *Fusarium oxysporum* f. sp. *cubense* (Foc) tropical race 4 (TR4) and Foc Lineage VI from naturally infected banana material.

CAV number^a^	Host	TR4^b^	Lineage VI^c^	VCG^d^	DNA (ng/μL)^e^	Ct value^f^
3824	S. Ndizi^g^	-	+	01222	5.7 x10 ^-4^	31.9
3826	S. Ndizi	-	+	0124/22	5.9 x 10 ^-4^	31.8
3827	S. Ndizi	-	+	0124/22	9.4 x 10^-4^	31.2
3828	S. Ndizi	-	+	01222	6.7 x 10 ^-5^	32.3
3829	S. Ndizi	-	+	0124/22	1.5 x 10 ^-4^	34.4
3830	S. Ndizi	-	+	0124/8/22	0.32 x 10^0^	23.2
3831	S. Ndizi	-	+	0124/8/22	3.5 x 10 ^-5^	36.8
3832	S. Ndizi	-	+	0124/22	4.3 x 10^-2^	26.2
3833	S. Ndizi	-	+	0124/8/22	1.3 x 10 ^-3^	31.3
3834	S. Ndizi	-	+	0124/22	3 x 10 ^-5^	36.9
3877	Cavendish	+	-	01213/16	1.8 x 10 ^-2^	26.9
3878	Cavendish	+	-	01213/16	7.8 x 10 ^-4^	31.4
3881	Cavendish	+	-	01213/16	5.9 x 10 ^-3^	28.5
3882	Cavendish	+	-	01213/16	0.13 x 10^0^	24.3
3883	Cavendish	+	-	01213/16	6.5 x 10^−3^	28.4
3884	Cavendish	+	-	01213/16	8.0 x 10^−2^	24.7
3885	Cavendish	+	-	01213/16	2.1 x 10^−3^	30.0
3887	Cavendish	+	-	01213/16	4.4 x 10^−5^	35.7
3888	Cavendish	+	-	01213/16	2.1 x 10^−2^	26.6
3889	Cavendish	+	-	01213/16	4.1 x 10^−2^	25.7

^a^ Culture collection housed at the Department of Plant Pathology, Stellenbosch University, South Africa.

^b^ A qPCR assay specific for Foc TR4 was used to screen each sample in duplicate, based on melt curve analysis samples were designated as positive (+) or negative (-).

^c^ A qPCR assay specific for Foc Lineage VI was used to screen each sample in duplicate, based on melt curve analysis samples were designated as positive (+) or negative (-).

^d^ Vegetative compatibility group (VCG).

^e^ The average DNA quantified for each positive sample using a matrix matched standard curve.

^f^ The average cycle threshold (Ct) value for each positive sample using a matrix matched standard curve.

^g^Sukari Ndizi.

## Discussion

The accurate identification of different races of Foc in plants, soil and water is essential for the early detection, containment and management of banana Fusarium wilt. This is particularly true when highly virulent strains are introduced into new areas, and when different strains of Foc cause disease to the same banana variety. A significant concern to both Africans and Latin Americans was the detections of Foc TR4 in Mozambique [17] and Colombia [47], respectively, and its potential to spread to disease-free areas and neighbouring countries. Borders in southern Africa are porous, and people often move planting material and animals across. Foc TR4 can cause disease to Gros Michel, a popular sweet dessert banana variety planted in both Africa and Central America, which is also affected by Foc race 1. If Foc TR4 is not expected by growers, Gros Michel may eventually, and unknowingly, serve as a source of infection of economically more important varieties, such as Cavendish bananas, which are highly susceptible to Foc TR4 but resistant to Foc races 1 and 2.

This study presents new primer sets for the quantitative detection of Foc Lineage VI, TR4 and STR4; thus all strains of the Fusarium wilt fungus found in Africa; with the exception of Foc VCG 01214, which was found in a small area in Malawi [48]. The primer sets can also detect strains of the same lineage and races collected outside Africa. Primers developed for VCG 01213/16 were specific to Foc TR4 and did not detect false positives previously reported for other TR4 primers by Magdama *et al*. (2019) [49] (results not presented). It also did not amplify other Foc strains causing disease to Cavendish bananas in the tropics, such as the closely related Foc VCG 0121 [31]. The Foc Lineage VI and TR4 primers were robust and specific, and accurately identified strains from Africa, Asia and Latin America by qPCR. The Foc STR4 primers, however, amplified DNA from non-target isolates. These include non-pathogenic *Fusarium oxysporum*, *F*. *proliferatum* and *F*. *saccharri*; all *Fusarium* species that have been found to occur in banana plant tissue before and are ubiquitous in the soil [50, 51]. To improve the specificity of the STR4 qPCR, a multiplex assay was designed with primers to target a *SIX9a* homolog that is present in all Foc isolates [39]. In the multiplex target DNA produces two distinct peaks in the post-amplification melt curve, which lowers the risk of detecting false positives. There is however often a degree of amplification bias whereby conditions favour one primer set over the other [52, 53]. The STR4 multiplex can only be used for qualitative detection directly from environmental samples. Absolute quantification, however is possible when no other Foc are present in a sample.

The LOQ and LOD of the optimised qPCR assays were similar to those achieved by [31, 32, 54] (10^−3^–10^−5^ ng/μL), which showed that the primers sets developed in this study were sensitive and suitable for the absolute quantification of Foc in complex environmental samples. The LOQ and LODs were higher in water than in plant material and soil, which implies that the Foc inoculum in environmental water needs to be concentrated by ultrafiltration or baiting if it is to be quantified. Extracting Foc DNA from water will also be a challenge due to the low spore concentrations and large sample volumes required for accurately detecting fungal pathogens [55, 56].

The identification of banana plants with Fusarium wilt symptoms in areas where Foc has not yet been detected is vital to track the spread of the disease, highlight weaknesses in containment and provide surrounding areas with an early warning system. The danger that seemingly healthy plants may also harbour Foc and spread banana Fusarium wilt to new areas is also of great concern [48]. Farmers and quarantine officials can be trained to identify Fusarium wilt symptoms and have symptomatic material tested with the developed qPCR assays. Detection of Foc from asymptomatic material using PCR or qPCR is, however, not recommended as the amount of pathogen inoculum might be lower than the LOD of the designed assays. Quantitative detection of Foc in banana plants can potentially also be used for greenhouse resistance screening. Studies by Vandemark and Barker [57] and Markakis *et al*. [58] on *Phytophthora medicaginis* in alfalfa and chickpea and *Verticillium* spp. in olives, respectively, showed that significantly more pathogen DNA was detected in susceptible than resistant hosts.

The developed soil qPCR assays proved robust for quantifying target DNA in inoculated soil samples. In practice, however, estimating pathogen inoculum from a field of soil is challenging as inoculum is often non-randomly distributed [59]. Sampling strategy is, therefore, paramount to ensure successful detection in soil. The number of samples, sample size, location and sample processing steps effect downstream results such as DNA quantity and reproducibility [60, 61]. Co-extracting environmental inhibitors with fungal DNA may also decrease amplification efficiency, resulting in an underestimation of pathogen inoculum levels or result in false negatives. Yet, Ophel-Keller *et al*. (2008) [62] developed a routine DNA-based testing service for the detection of several soil-borne pathogens by qPCR to predict the extent of losses expected prior to planting. The quantitative detection of pathogens like Foc in soil could be used to evaluate soil inoculum levels necessary for disease and develop a model similar to that of Ophel-Keller *et al*. (2008) [62]. The reproducibility and repeatability of both sampling strategies and DNA extraction methods should however, be accurately determined to ensure reliability.

The cost per sample, and ease of use, are important considerations for the adoption of any detection tool, especially in developing countries. Loop-mediated isothermal amplification (LAMP) has been developed for quantitative detection of Foc TR4 [33], which offers some advantages for the quantitative detection of Foc, as the machine is portable and easy to use. The assay is, however, reliant on a Genie II machine, which is often neither available nor affordable to most research stations and quarantine units in Africa, while chemistry and specific plastics are more expensive than those used for qPCR. The available qPCR assay for Foc TR4 detection [31] utilizes a hydrolysis probe, which is considered more specific, but also more expensive [63], than intercalating dyes. Assays based on SYBR® green used in the current study, therefore, offers a more affordable option and the post-analysis melt curve could be utilised to evaluate specificity [64].

## Conclusion

Foc Lineage VI, TR4 and STR4 markers were developed for qPCR assays that can quantify target pathogens in plant, water and soil samples. The assays were designed with the availability and affordability of equipment and the cost per sample in mind. A large set of isolates representing the full diversity of Foc and selected *Fusarium* spp. was used to evaluate marker specificity. Specificity was maintained when Foc was measured in artificially inoculated plant, water and soil samples. To detect Foc in naturally infected water and soil samples, the further optimisation and modified sampling strategies to concentrate and homogenise collected samples are required.

## Supporting information

S1 Fig Three pipelines to illustrate the strategy employed to design primers specific to *Fusarium oxysporum* f. sp. *cubense* (Foc) Lineage VI, Foc Tropical Race 4 (VCG 01213/16) and Foc Subtropical Race 4 (VCG 0120/15). (PPTX)

S2 Fig Sequences of amplicons and location of matches to Foc II5 reference genome for primer sets designed in the study. (PPTX)

S1 Appendix Multiple sequence alignment of the DNA-directed RNA polymerase III subunit beta (RPC2) gene region employed to design primers specific to *Fusarium oxysprum* f. sp. *cubense* Lineage VI (VCG 0124/8/12/20/22). (FAS)

S2 Appendix Multiple sequence alignment of the FOIG 03031 hypothetical protein region employed to design primers specific to *Fusarium oxysprum* f. sp. *cubense* subtropical race 4 VCG 0120/15. (FAS)

S3 Appendix Multiple sequence alignment of a non-coding in supercontig KK036133 of the Foc II5 reference genome employed to design primers specific to *Fusarium oxysprum* f. sp. *cubense* tropical race 4 VCG 01213/16. (FAS)

S4 Appendix Multiple sequence alignment of the *secreted-in-xylem (SIX)9* homolog employed to design primers specific to all *Fusarium oxysprum* f. sp. *cubense* isolates. (FAS)

S1 Table The reproducibility of DNA quantification, based on quantitative PCR, from plant water and soil samples inoculated with different *Fusarium oxysporum* f. sp. *cubense* isolates. (DOCX)

S2 Table The repeatability of DNA quantification, based on quantitative PCR, from plant water and soil samples inoculated with different *Fusarium oxysporum* f. sp. *cubense* isolates. (DOCX)

S3 Table Analysis of variances of the different DNA extraction methods to isolate *Fusarium oxysporum* f. sp. *cubense* DNA from environmental samples. (DOCX)

S4 Table The reproducibility of the qPCR assays quantifying *Fusarium oxysporum* f. sp. cubense in plant, water and soil samples. (DOCX)

S5 Table The repeatability of qPCR assays quantifying *Fusarium oxysporum* f. sp. *cubense* in plant, water and soil samples. (DOCX)
