# Supplementary material for: Quantitative detection of economically important Fusarium oxysporum f. sp. cubense strains in Africa in plants, soil and water
Source: PLoS One. 2020 Jul 20;15(7):e0236110. doi: 10.1371/journal.pone.0236110 (PMC7371176; doi:10.1371/journal.pone.0236110)
Supplement: S1 Fig — (PPTX) [file pone.0236110.s001.pptx]

## Slide 1
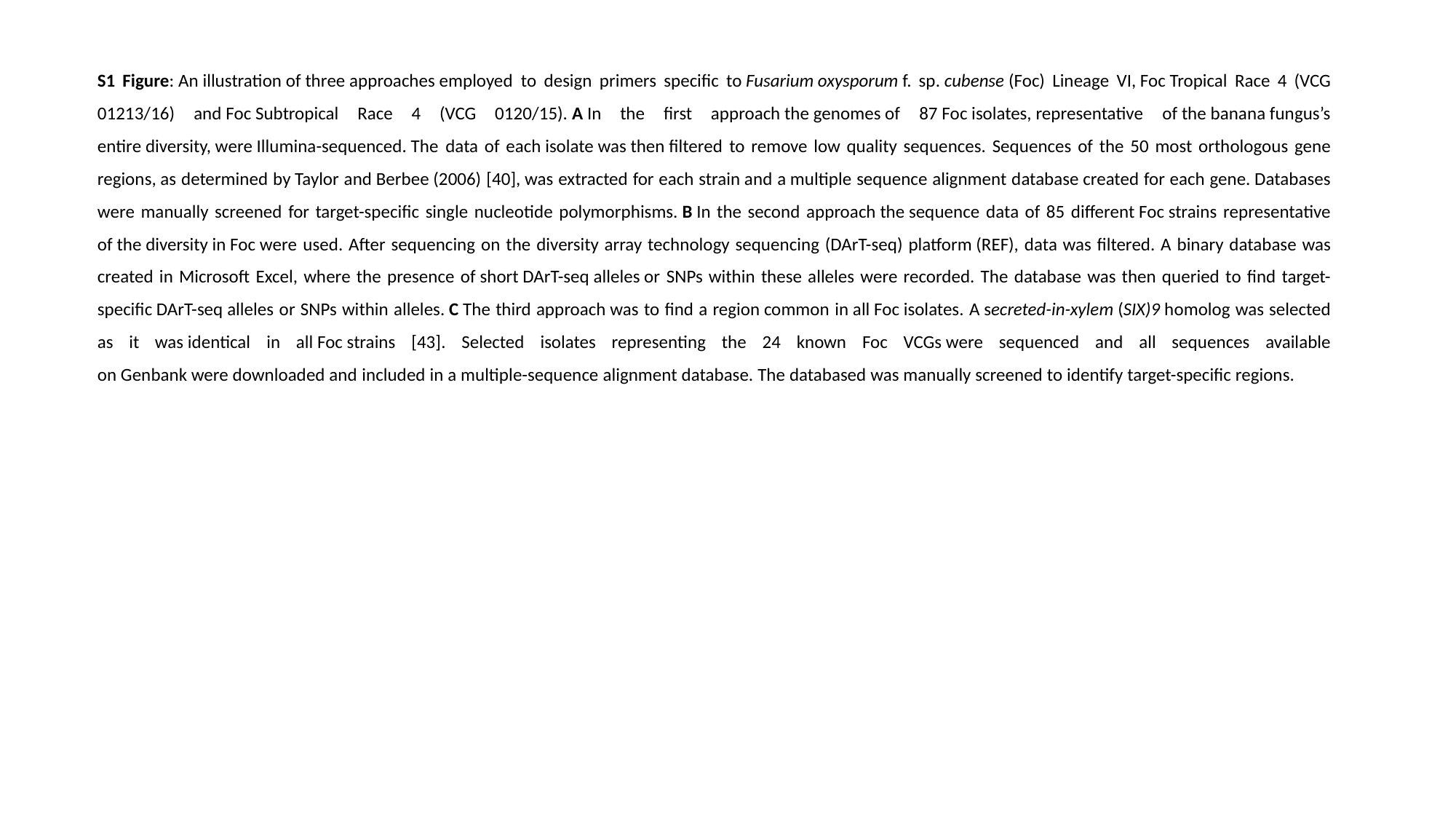

S1 Figure: An illustration of three approaches employed to design primers specific to Fusarium oxysporum f. sp. cubense (Foc) Lineage VI, Foc Tropical Race 4 (VCG 01213/16) and Foc Subtropical Race 4 (VCG 0120/15). A In the first approach the genomes of 87 Foc isolates, representative of the banana fungus’s entire diversity, were Illumina-sequenced. The data of each isolate was then filtered to remove low quality sequences. Sequences of the 50 most orthologous gene regions, as determined by Taylor and Berbee (2006) [40], was extracted for each strain and a multiple sequence alignment database created for each gene. Databases were manually screened for target-specific single nucleotide polymorphisms. B In the second approach the sequence data of 85 different Foc strains representative of the diversity in Foc were used. After sequencing on the diversity array technology sequencing (DArT-seq) platform (REF), data was filtered. A binary database was created in Microsoft Excel, where the presence of short DArT-seq alleles or SNPs within these alleles were recorded. The database was then queried to find target-specific DArT-seq alleles or SNPs within alleles. C The third approach was to find a region common in all Foc isolates. A secreted-in-xylem (SIX)9 homolog was selected as it was identical in all Foc strains [43]. Selected isolates representing the 24 known Foc VCGs were sequenced and all sequences available on Genbank were downloaded and included in a multiple-sequence alignment database. The databased was manually screened to identify target-specific regions.

## Slide 2
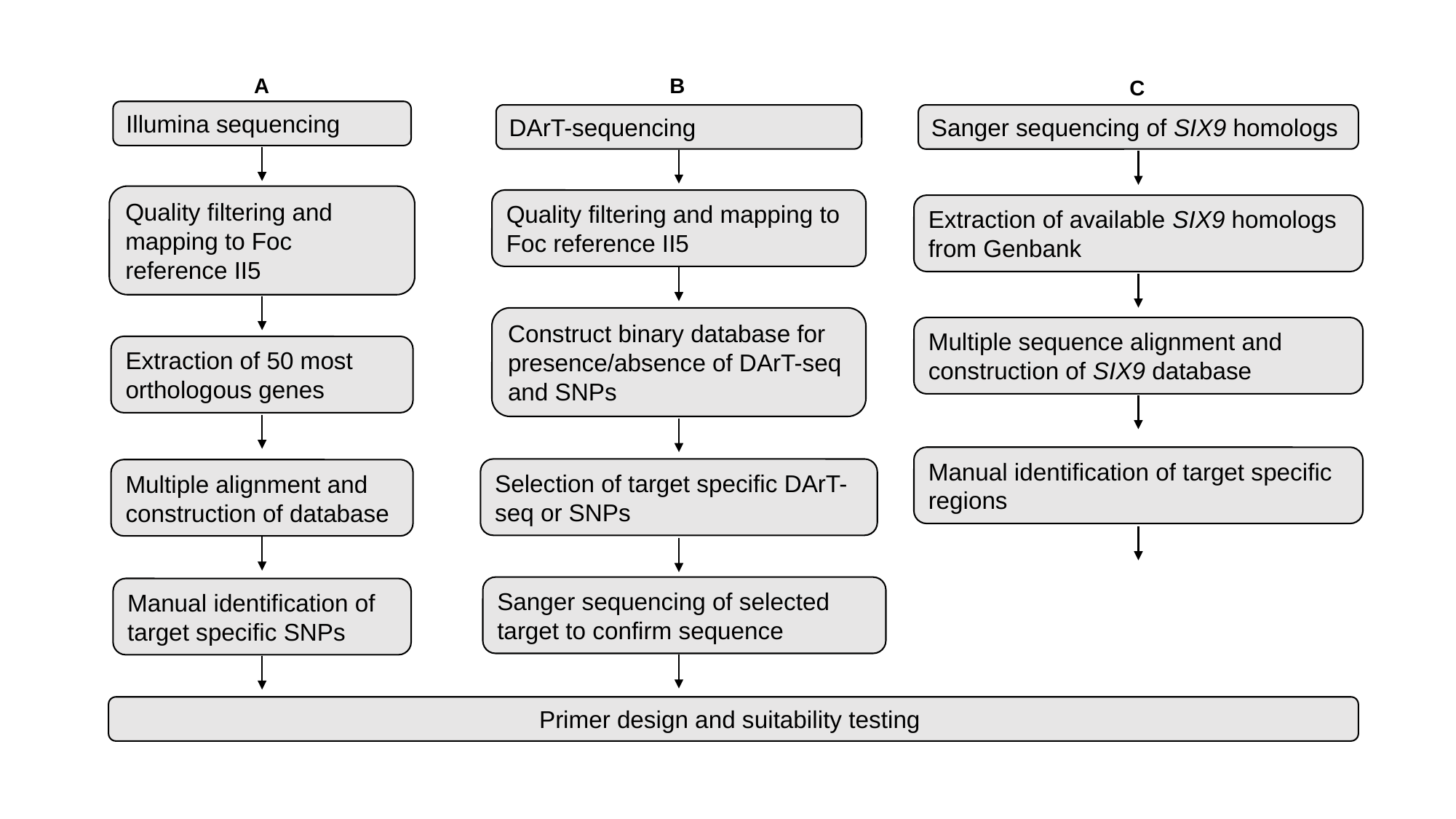

B
DArT-sequencing
Quality filtering and mapping to Foc reference II5
Construct binary database for presence/absence of DArT-seq and SNPs
Selection of target specific DArT-seq or SNPs
Sanger sequencing of selected target to confirm sequence
A
Illumina sequencing
Quality filtering and mapping to Foc reference II5
Extraction of 50 most orthologous genes
Multiple alignment and construction of database
Manual identification of target specific SNPs
C
Sanger sequencing of SIX9 homologs
Extraction of available SIX9 homologs from Genbank
Multiple sequence alignment and construction of SIX9 database
Manual identification of target specific regions
Primer design and suitability testing
