# Supplementary material for: Quantitative detection of economically important Fusarium oxysporum f. sp. cubense strains in Africa in plants, soil and water
Source: PLoS One. 2020 Jul 20;15(7):e0236110. doi: 10.1371/journal.pone.0236110 (PMC7371176; doi:10.1371/journal.pone.0236110)
Supplement: S2 Fig — (PPTX) [file pone.0236110.s002.pptx]

## Slide 1
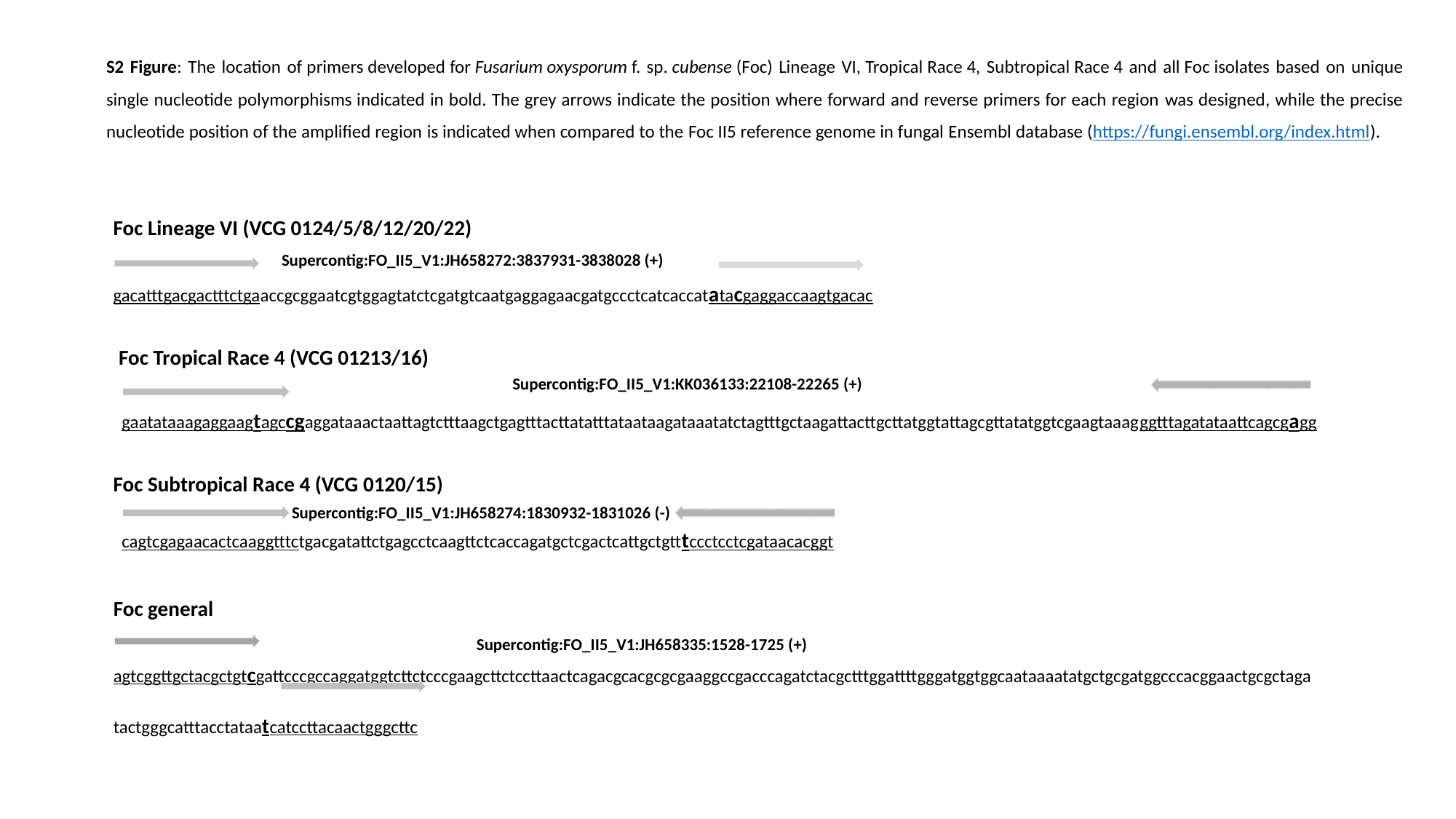

S2 Figure: The location of primers developed for Fusarium oxysporum f. sp. cubense (Foc) Lineage VI, Tropical Race 4, Subtropical Race 4 and all Foc isolates based on unique single nucleotide polymorphisms indicated in bold. The grey arrows indicate the position where forward and reverse primers for each region was designed, while the precise nucleotide position of the amplified region is indicated when compared to the Foc II5 reference genome in fungal Ensembl database (https://fungi.ensembl.org/index.html).
Foc Lineage VI (VCG 0124/5/8/12/20/22)
Supercontig:FO_II5_V1:JH658272:3837931-3838028 (+)
gacatttgacgactttctgaaccgcggaatcgtggagtatctcgatgtcaatgaggagaacgatgccctcatcaccatatacgaggaccaagtgacac
Foc Tropical Race 4 (VCG 01213/16)
gaatataaagaggaagtagccgaggataaactaattagtctttaagctgagtttacttatatttataataagataaatatctagtttgctaagattacttgcttatggtattagcgttatatggtcgaagtaaagggtttagatataattcagcgagg
Foc Subtropical Race 4 (VCG 0120/15)
Supercontig:FO_II5_V1:JH658274:1830932-1831026 (-)
cagtcgagaacactcaaggtttctgacgatattctgagcctcaagttctcaccagatgctcgactcattgctgtttccctcctcgataacacggt
Foc general
Supercontig:FO_II5_V1:JH658335:1528-1725 (+)
agtcggttgctacgctgtcgattcccgccaggatggtcttctcccgaagcttctccttaactcagacgcacgcgcgaaggccgacccagatctacgctttggattttgggatggtggcaataaaatatgctgcgatggcccacggaactgcgctagatactgggcatttacctataatcatccttacaactgggcttc
Supercontig:FO_II5_V1:KK036133:22108-22265 (+)
