# Supplementary material for: Quantitative detection of economically important Fusarium oxysporum f. sp. cubense strains in Africa in plants, soil and water
Source: PLoS One. 2020 Jul 20;15(7):e0236110. doi: 10.1371/journal.pone.0236110 (PMC7371176; doi:10.1371/journal.pone.0236110)
Supplement: S2 Table — (DOCX) [file pone.0236110.s008.docx]

**S2 Table.** The repeatability of DNA quantification, based on quantitative PCR, from plant water and soil samples inoculated with different *Fusarium oxysporum* f. sp. *cubense* isolates (Foc).

|  |  | Day 1^b^ | | Day 2 | |
| --- | --- | --- | --- | --- | --- |
|  |  | Target DNA concentration (ng/µL)^c^ | SD of target DNA^e^ | Target DNA concentration (ng/µL) | SD of target DNA |
| Plant^a^ | CAV 188 | 0.003 | 0.002 | 0.005 | 0.002 |
|  | CAV 184 | 0.003 | 0.001 | 0.008 | 0.004 |
|  | CAV 2400 | 0.025 | 0.006 | 0.011* | 0.004* |
|  | NRRL 36117 | 0.039 | 0.027 | 0.054 | 0.014 |
| Water | CAV 188 | 0.066 | 0.021 | 0.035 | 0.010 |
|  | CAV 184 | 0.088 | 0.009 | 0.056* | 0.021* |
|  | CAV 2400 | 0.266 | 0.086 | 0.352 | 0.036 |
|  | NRRL 36117 | 0.354 | 0.008 | 0.345 | 0.028 |
| Soil | CAV 188 | 0.038 | 0.016 | 0.060 | 0.039 |
|  | CAV 184 | 0.060* | 0.022* | 0.046 | 0.022 |
|  | CAV 2400 | 0.087 | 0.014 | 0.041 | 0.018 |
|  | NRRL 36117 | 0.020 | 0.002 | 0.014 | 0.002 |

SD – Standard deviation

^a^ Environmental sample type (plant/water/soil) infected with Foc Lineage VI isolates (CAV 188, 184, 2400 or NRRL 36117).

^b^ The day on which the target DNA was extracted.

^c^ The average DNA concentration of three or six (*) sub-samples measured with qPCR.

^e^ The standard deviation between target DNA concentrations from three or six (*) sub-samples.
