# Supplementary material for: Quantitative detection of economically important Fusarium oxysporum f. sp. cubense strains in Africa in plants, soil and water
Source: PLoS One. 2020 Jul 20;15(7):e0236110. doi: 10.1371/journal.pone.0236110 (PMC7371176; doi:10.1371/journal.pone.0236110)
Supplement: S3 Table — (DOCX) [file pone.0236110.s009.docx]

**S3 Table.** Analysis of variances of the different extraction methods to isolate *Fusarium oxysporum* f. sp. *cubense* DNA from environmental samples.

|  | Nucleospin® Plant II minipreps | | AMpure water extraction | | Nucleospin® Soil extraction | |
| --- | --- | --- | --- | --- | --- | --- |
| Sources of variation | DF^a^ | Pr > F^b^ | DF | Pr > F | DF | Pr > F |
| Day of the DNA extraction ^c^ | 1 | 0.8903 | 1 | 0.1370 | 1 | 0.0790 |
| Lineage VI isolate^d^ | 3 | <.0001 | 3 | 0.0057 | 3 | <.0001 |
| Day x Lineage VI isolate^e^ | 3 | 0.0907 | 3 | 0.0782 | 3 | 0.0207 |

^a^Degrees of freedom (DF).

^b^The significance probability (P) value associated with the F-Value.

^c^The day of extraction (1 or 2).

^d^Lineage VI isolate the environmental sample (plant/water soil) was infected with (CAV 184/CAV 188/CAV 2400/ NRRL 36117).

^e^The interaction between the day of the DNA extraction and the Lineage VI isolate which infected the sample.
