# Supplementary material for: Quantitative detection of economically important Fusarium oxysporum f. sp. cubense strains in Africa in plants, soil and water
Source: PLoS One. 2020 Jul 20;15(7):e0236110. doi: 10.1371/journal.pone.0236110 (PMC7371176; doi:10.1371/journal.pone.0236110)
Supplement: S4 Table — (DOCX) [file pone.0236110.s010.docx]

**S4 Table.** The reproducibility of the qPCR assays quantifying *Fusarium oxysporum* f. sp. cubense in plant, water and soil samples.

|  |  | Lineage VI Ct values | | | TR4 Ct values | | | STR4 Ct values | | |
| --- | --- | --- | --- | --- | --- | --- | --- | --- | --- | --- |
|  |  | A1^b^ | A2 | SD^c^ | A1 | A2 | SD | A1 | A2 | SD |
| Plant^a^ | Isolate 1 | 31.04^d^ | 31.39 | 0.295 | 16.99 | 16.87 | 0.087 | 24.77 | 24.63 | 0.161 |
|  | Isolate 2 | 31.47 | 31.98* | 0.342 | 21.36 | 21.57 | 0.153 | 20.48 | 19.53* | 0.455 |
|  | Isolate 3 | 27.85 | 27.57 | 0.301 | 31.29 | 31.56* | 0.194 | 25.54 | 24.84 | 0.400 |
| Water | Isolate 1 | 24.62 | 24.47 | 0.106 | 18.30 | 18.39 | 0.066 | 19.67 | 19.58 | 0.177 |
|  | Isolate 2 | 23.59 | 24.03 | 0.336 | 17.77 | 17.75* | 0.018 | 22.39 | 22.48 | 0.140 |
|  | Isolate 3 | 21.39 | 21.42* | 0.021 | 24.94 | 24.76 | 0.123 | 26.40* | 26.47 | 0.141 |
| Soil | Isolate 1 | 29.51 | 29.24 | 0.323 | 15.84 | 15.71 | 0.117 | 27.57* | 27.36 | 0.274 |
|  | Isolate 2 | 28.10* | 27.73 | 0.239 | 14.34 | 14.32 | 0.014 | 21.36 | 21.27 | 0.055 |
|  | Isolate 3 | 32.42 | 32.25 | 0.281 | 24.32* | 24.73 | 0.223 | 25.71 | 25.55 | 0.119 |

Ct values – Cycle threshold value according to qPCR analyses.SD – Standard deviation

^a^The environmental sample type (plant, water or soil) infected with three different positive isolates; CAV 184, CAV 188 and CAV 2400 for Lineage VI, CAV 789, CAV 3326 and CAV 3049 for TR4 and CAV 92, CAV 191 and CAV 612 for STR4.

^b^The qPCR assay in which DNA, extracted from the target isolates, was analysed in triplicate.

^c^The standard deviation between average Ct values between duplicate qPCR assay.

^d^The average Ct of three or six (*) technical replicates.
