# Supplementary material for: Quantitative detection of economically important Fusarium oxysporum f. sp. cubense strains in Africa in plants, soil and water
Source: PLoS One. 2020 Jul 20;15(7):e0236110. doi: 10.1371/journal.pone.0236110 (PMC7371176; doi:10.1371/journal.pone.0236110)
Supplement: S5 Table — (DOCX) [file pone.0236110.s011.docx]

**S5 Table.** The repeatability of qPCR assays quantifying *Fusarium oxysporum* f. sp. *cubense* (Foc) in plant, water and soil samples.

|  | |  | Cycle Threshold values | | Standard deviations | |
| --- | --- | --- | --- | --- | --- | --- |
|  | |  | High^b^ | Low^c^ | SD_H_^d^ | SD_L_^e^ |
| Plant^a^ | Lineage VI | | 31.98* | 27.57 | 0.136 | 0.228 |
|  | TR4 | | 31.56* | 16.87 | 0.248 | 0.021 |
|  | STR4 | | 25.54 | 19.53* | 0.070 | 0.180 |
| Water | Lineage VI | | 24.62 | 21.39 | 0.146 | 0.125 |
|  | TR4 | | 25.00 | 17.75* | 0.268 | 0.083 |
|  | STR4 | | 26.48 | 19.58 | 0.133 | 0.180 |
| Soil | Lineage VI | | 32.42 | 27.73 | 0.279 | 0.284 |
|  | TR4 | | 24.73* | 15.71 | 0.091 | 0.070 |
|  | STR4 | | 27.57 | 21.27 | 0.276 | 0.032 |

SD – Standard deviation

^a^The environmental sample type (plant, water or soil).

^b^The highest mean Ct value of target (Lineage VI/TR4/STR4) DNA in an environment with three or six replicates (*).

^c^The lowest mean Ct value of target (Lineage VI/TR4/STR4) DNA in an environment with three or six replicates (*).

^d^The standard deviation between Ct values from the sample with the highest mean CT.

^e^The standard deviation between Ct values from the sample with the lowest mean CT.
